# Supplementary material for: Implications of Endogenous Small Regulatory RNAs on Gene Silencing in Mollusks
Source: bioRxiv. 2025 May 23:2025.05.19.654968. Preprint. [Version 1] doi: 10.1101/2025.05.19.654968 (PMC12139959; doi:10.1101/2025.05.19.654968)
Supplement: 2 [file NIHPP2025.05.19.654968v1-supplement-2.pdf]

bioRxiv preprint doi: https://doi.org/10.1101/2025.05.19.654968; this version posted May 23, 2025. The copyright holder for this preprint (which was not certified by peer review) is the author/funder, who has granted bioRxiv a license to display the preprint in perpetuity. It is made available under aCC-BY 4.0 International license.

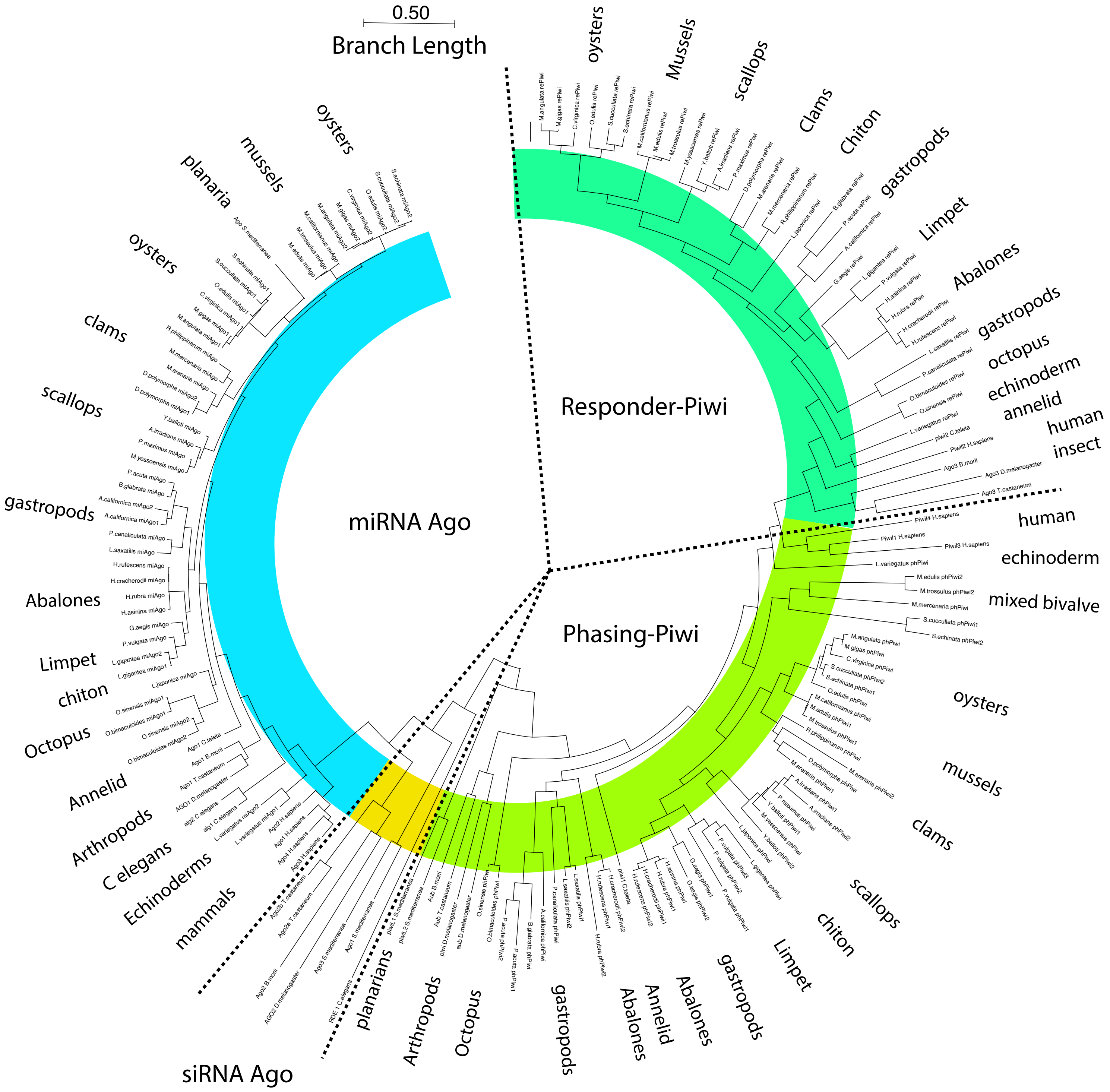

Supplementary Figure 1: Phylogeny Analysis of AGO/PIWI Proteins in Mollusca.
Comprehensive tree showing phylogenetic relationship among the AGO/PIWI proteins of different species. While numerous miAGO (blue), phPiwi (lime green), and rePiwi (pastel green) were identified in Mollusca, siAGO (yellow) was only identified in outgroup reference species: *D. melanogaster*, *B. morii*, *S. mediterranea*, *T. castaneum*, and *C. elegans*.

bioRxiv preprint doi: https://doi.org/10.1101/2025.05.19.654968; this version posted May 23, 2025. The copyright holder for this preprint (which was not certified by peer review) is the author/funder, who has granted bioRxiv a license to display the preprint in perpetuity. It is made available under aCC-BY 4.0 International license.

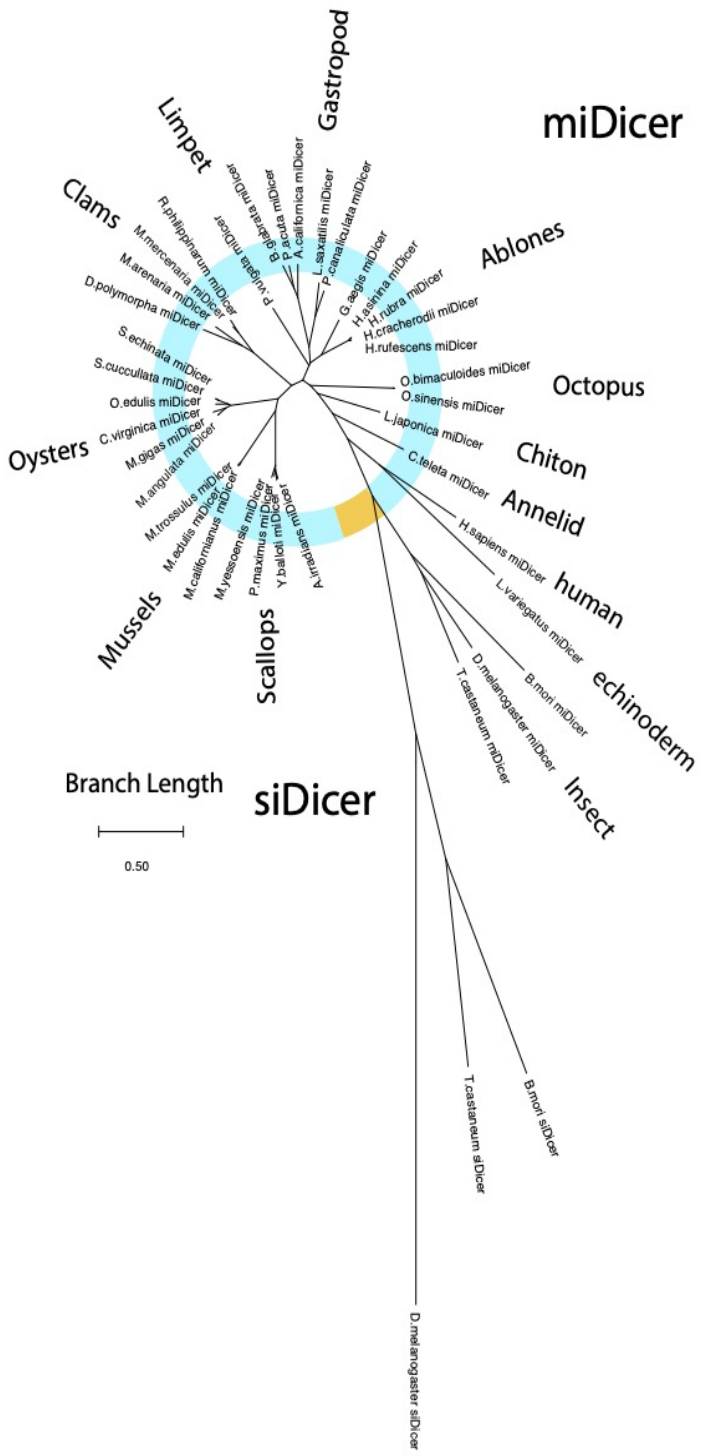

Supplementary Figure 2: Phylogeny Analysis of Dicer Proteins in Mollusca. Phylogenetic relationship among the Dicer proteins of different species. The Dicer1 reference species (blue) are *L. stagnalis*, *C. teleta*, *H. sapiens*, *D. melanogaster*, and *C. elegans*. The siDicer reference species (yellow) are *D. melanogaster*, *Tribolium castaneum*, and *Bombyx mori*. Phylogenetic analysis demonstrates a correlation between all known Mollusca Dicer and known miDicer proteins rather than a correlation with siDicer proteins.

bioRxiv preprint doi: https://doi.org/10.1101/2025.05.19.654968; this version posted May 23, 2025. The copyright holder for this preprint (which was not certified by peer review) is the author/funder, who has granted bioRxiv a license to display the preprint in perpetuity. It is made available under aCC-BY 4.0 International license.

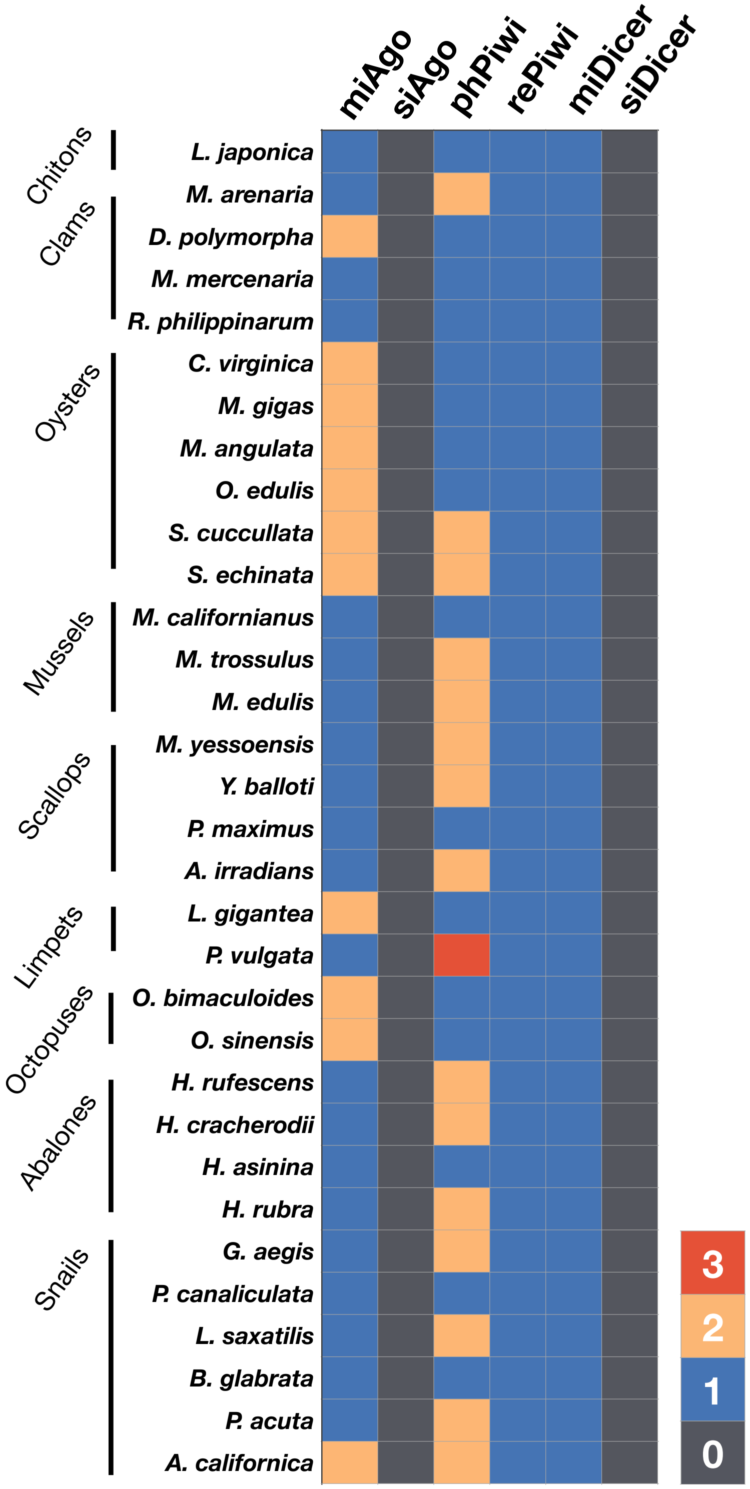

Supplementary Figure 3: sRNA proteins, siRNAs, and miRNAs in Mollusca. An overview of sRNA-associated proteins in mollusks, sorted phylogenetically. While numerous Ago and Piwi proteins were identified, neither siAgo nor siDicer proteins were observed.

bioRxiv preprint doi: https://doi.org/10.1101/2025.05.19.654968; this version posted May 23, 2025. The copyright holder for this preprint (which was not certified by peer review) is the author/funder, who has granted bioRxiv a license to display the preprint in perpetuity. It is made available under aCC-BY 4.0 International license.

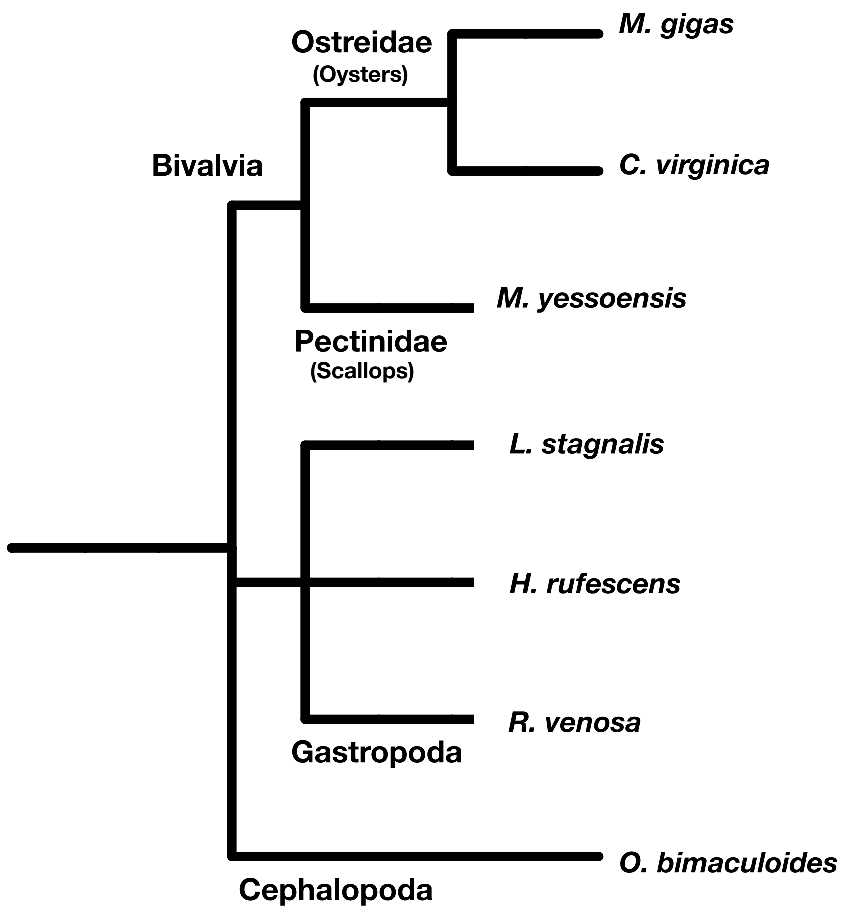

Supplementary Figure 4: Phylogeny of Mollusca Analyzed. Phylogenetic tree within the Mollusca phylum indicating the species whose sRNA-seq data we examined in this project: *M. gigas*, *C. virginica*, *M. yessoensis*, *L. stagnalis*, *H. rufescens, R. venosa*, and *O. bimaculoides*.

bioRxiv preprint doi: https://doi.org/10.1101/2025.05.19.654968; this version posted May 23, 2025. The copyright holder for this preprint (which was not certified by peer review) is the author/funder, who has granted bioRxiv a license to display the preprint in perpetuity. It is made available under aCC-BY 4.0 International license.

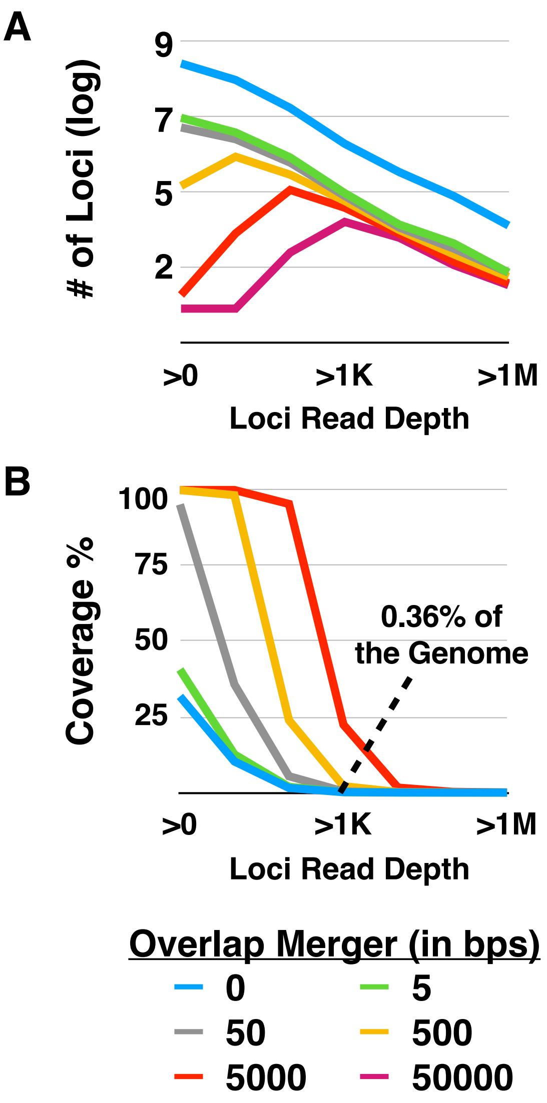

Supplementary Figure 5. sRNA Thresholding sRNA of *Crassostrea virginica*. An optimal genome coverage threshold was determined by plotting loci read depth compared to the merging of read overlaps. **(A)** The number of loci (in log) identified when sequential overlap mergers (lines) were plotted against an increasing minimum of read depths per loci (x axis). **(B)** The loci's total genomic coverage when sequential overlap mergers (lines) were plotted against an increasing minimum of read depths per loci (x axis). Loci that were merged with other regions of interest within 500 bp (yellow line) and had a minimum read depth of 1,000 appear to represent the median in both the number of loci present and overall genomic coverage.

bioRxiv preprint doi: https://doi.org/10.1101/2025.05.19.654968; this version posted May 23, 2025. The copyright holder for this preprint (which was not certified by peer review) is the author/funder, who has granted bioRxiv a license to display the preprint in perpetuity. It is made available under aCC-BY 4.0 International license.

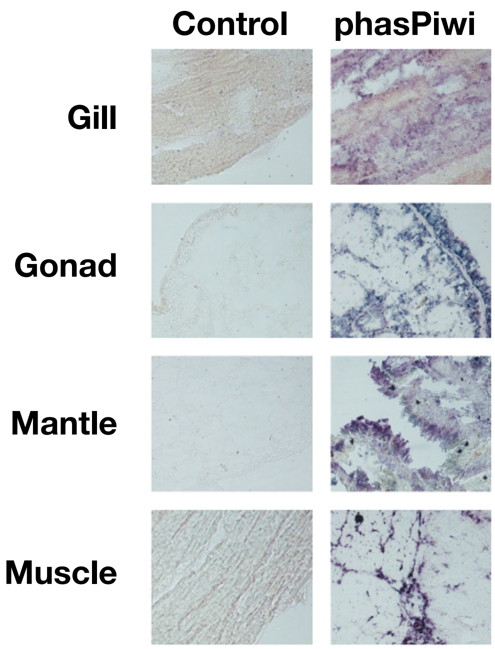

Supplementary Figure 6: *In Situ* Hybridization of *C. virginica*. Visualization of phas-piRNAs in various *C. virginica* tissue types. The purple represents piRNAs found in cells within each tissue.

bioRxiv preprint doi: https://doi.org/10.1101/2025.05.19.654968; this version posted May 23, 2025. The copyright holder for this preprint (which was not certified by peer review) is the author/funder, who has granted bioRxiv a license to display the preprint in perpetuity. It is made available under aCC-BY 4.0 International license.

Species	Genome Assembly	GenBank Assembly
Aplysia californica	AplCal3.0	GCA_000002075.2
Argopecten irradians	Ai_NY	GCA_041381155.1
Biomphalaria glabrata	xgBioGlab47.1	GCA_947242115.1
Crassostrea virginica	C_virginica-3.0	GCA_002022765.4
Dreissena polymorpha	UMN_Dpol_1.0	GCA_020536995.1
Gigantopelta aegis	Gae_host_genome	GCA_016097555.1
Haliotis asinina	JCU_Hal_asi_v2	GCA_037392515.2
Haliotis cracherodii	xgHalCrac1.p	GCA_022045235.1
Haliotis rubra	ASM391887v1	GCA_003918875.1
Haliotis rufescens	xgHalRufe1.0.p	GCA_023055435.1
Liolophura japonica	CUHK_Ljap_v2	GCA_032854445.2
Littorina saxatilis	US_GU_Lsax_2.0	GCA_037325665.1
Lottia gigantea	Helro1	GCA_000327385.1
Lytechinus variegatus	Lvar_3.0	GCA_018143015.1
Magallana angulata	ASM2561291v2	GCA_025612915.2
Magallana gigas	xbMagGiga1.1	GCA_963853765.1
Mercenaria mercenaria	MADL_Memer_1	GCA_021730395.1
Mizuhopecten yessoensis	ASM211388v2	GCA_002113885.2
Mya arenaria	ASM2691426v1	GCA_026914265.1
Mytilus californianus	xbMytCali1.0.p	GCA_021869535.1
Mytilus edulis	xbMytEdul2.2	GCA_963676685.2
Mytilus trossulus	PNRI_Mtr1.2.1.hap1	GCA_036588685.2
Octopus bimaculoides	ASM119413v2	GCA_001194135.2
Octopus sinensis	ASM634580v1	GCA_006345805.1
Ostrea edulis	xbOstEdul1.1	GCA_947568905.1
Patella vulgata	xgPatVulg1.2	GCA_932274485.2
Pecten maximus	xPecMax1.1	GCA_902652985.1
Physella acuta	ASM2847654v3	GCA_028476545.3
Pomacea canaliculata	ASM307304v1	GCA_003073045.1
Rapana venosa	ASM2875187v1	GCA_028751875.1
Ruditapes philippinarum	ASM2657151v2	GCA_026571515.2
Saccostrea cuccullata	CSIRO_AGI_Scuc_v1	GCA_032062105.1
Saccostrea echinata	CSIRO_AGI_Sech_v1	GCA_033153115.1
Ylistrum balloti	AGI_CSIRO_Ybal_v1	GCA_031769215.1

Supplemental Table 1. Overview of Genomes. Summary of NCBI Mollusca (and *L. variegatus*) genomes used in data computation.

bioRxiv preprint doi: https://doi.org/10.1101/2025.05.19.654968; this version posted May 23, 2025. The copyright holder for this preprint (which was not certified by peer review) is the author/funder, who has granted bioRxiv a license to display the preprint in perpetuity. It is made available under aCC-BY 4.0 International license.

Species	Tissue	SRA	Reads
C. virginica	INH_Male_Gonad	SRR33418593	51703409
C. virginica	INH_Female1_Gonad	SRR33418592	59187962
C. virginica	INH_Female2_Gonad	SRR33418581	50650416
C. virginica	INH_Child1B	SRR33418570	49392863
C. virginica	INH_Child1C	SRR33418559	52729896
C. virginica	INH_Child1E	SRR33418549	49693994
C. virginica	INH_Child2F	SRR33418548	42504756
C. virginica	INH_Child2H	SRR33418547	52188423
C. virginica	INH_Child2I	SRR33418546	49327186
C. virginica	AdductorMuscle1	SRR33418545	9448493
C. virginica	AdductorMuscle2	SRR33418591	9348118
C. virginica	AdductorMuscle3	SRR33418590	9665186
C. virginica	AdductorMuscle4	SRR33418589	9419443
C. virginica	AdductorMuscle9	SRR33418588	9807973
C. virginica	AdductorMuscle10	SRR33418587	9593441
C. virginica	AdductorMuscle11	SRR33418586	9923013
C. virginica	AdductorMuscle12	SRR33418585	9571981
C. virginica	AdductorMuscle13	SRR33418584	9732943
C. virginica	AdductorMuscle14	SRR33418583	9476406
C. virginica	AdductorMuscle15	SRR33418582	9482977
C. virginica	AdductorMuscle16	SRR33418580	9271958
C. virginica	Gill1	SRR33418579	14050252
C. virginica	Gill2	SRR33418578	13712068
C. virginica	Gill3	SRR33418577	14609013
C. virginica	Gill4	SRR33418576	14100857
C. virginica	Gonad1	SRR33418575	13244351
C. virginica	Gonad2	SRR33418574	12940268
C. virginica	Gonad3	SRR33418573	13617227
C. virginica	Gonad4	SRR33418572	13226919
C. virginica	CvirMantleSnRNA_1	SRR33450334	41392647
C. virginica	CvirMantleSnRNA_2	SRR33450334	41392647
R. venosa	Pre-Competent_Larvae1	SRR5931826	10157033
R. venosa	Pre-Competent_Larvae2	SRR5931827	10827765
R. venosa	Pre-Competent_Larvae3	SRR5931828	10601339
R. venosa	Competent_Larvae1	SRR5931829	12156108
R. venosa	Competent_Larvae2	SRR5931830	11451480
R. venosa	Competent_Larvae3	SRR5931831	10023123
R. venosa	Post-Larvae1	SRR5931832	10678504
R. venosa	Post-Larvae2	SRR5931833	11622298
R. venosa	Post-Larvae3	SRR5931834	12087773
M. yessoensis	SmoothMuscle1	SRR18577892	10486504
M. yessoensis	SmoothMuscle2	SRR18577891	11914196
M. yessoensis	SmoothMuscle3	SRR18577890	10417121
M. yessoensis	StriatedMuscle1	SRR18577889	12011743
M. yessoensis	StriatedMuscle2	SRR18577888	14665041
M. yessoensis	StriatedMuscle3	SRR18577887	11196753
M. gigas	AdductorMuscle1	SRR6489635	27407026
M. gigas	AdductorMuscle2	SRR6489636	29698653
M. gigas	Gonad1	SRR6489633	28031758
M. gigas	Gonad2	SRR6489634	30366538
H rufescens	AdductorMuscle	SRR33418563	51801246
L. variegatus	Gonad_Ctrl1	SRR33418561	40639852
L. variegatus	Gonad_Ctrl2	SRR33418560	31498024
L. variegatus	Gonad_Ctrl3	SRR33418558	48932488
L. variegatus	Gonad_Reg1	SRR33418557	51016857
L. variegatus	Gonad_Reg2	SRR33418556	44795701
L. variegatus	Gonad_Reg3	SRR33418555	45765823
L. variegatus	INH_Male_Gonad	SRR33418554	136816008
L. variegatus	INH_Female1_Gonad	SRR33418553	131933301
L. variegatus	INH_Female2_Gonad	SRR33418552	115282154
L. variegatus	INH_ChildM1F1	SRR33418551	134202757
L. variegatus	INH_ChildM1F2	SRR33418550	187061029
O. bimaculoides	Tentacle_Ctrl1	SRR33418569	56141057
O. bimaculoides	Tentacle_Ctrl2	SRR33418568	50283992
O. bimaculoides	Tentacle_Ctrl3	SRR33418567	36235581
O. bimaculoides	Tentacle_Reg1	SRR33418566	21210311
O. bimaculoides	Tentacle_Reg2	SRR33418565	39412129
O. bimaculoides	Tentacle_Reg3	SRR33418564	41426978
D. melanogaster	Ovary_WT1	SRR11680898	39157763
D. melanogaster	Ovary_WT2	SRR11680899	31733032
D. melanogaster	Ovary_WT3	SRR11680900	34563946
D. melanogaster	Testes_WT1	SRR12313524	19163553
D. melanogaster	Testes_WT2	SRR12313525	16768121
D. melanogaster	Testes_WT3	SRR12313526	18081329
M. musculus	Ovary_Ctrl1	SRR13848060	20989881
M. musculus	Ovary_Ctrl3	SRR13848061	43772792
M. musculus	Ovary_Ctrl6	SRR13848062	26697670
M. musculus	Testis1	SRR24821465	7159893
M. musculus	Testis2	SRR24821466	9348548
M. musculus	Testis3	SRR24821469	9546916
M. musculus	Testis4	SRR24821470	10192831
S. mediterranea	Salami_Section10	SRR12426750	27373271
S. mediterranea	Salami_Section9	SRR12426751	7597498
S. mediterranea	Salami_Section8	SRR12426752	12112061
S. mediterranea	Salami_Section7	SRR12426753	9028748
S. mediterranea	Salami_Section6	SRR12426754	6048316
S. mediterranea	Salami_Section5	SRR12426755	8503488
S. mediterranea	Salami_Section4	SRR12426756	6511945
S. mediterranea	Salami_Section3	SRR12426757	11365797
S. mediterranea	Pharynx_Section12	SRR12426758	8309356
S. mediterranea	Salami_Section11	SRR12426759	8187802
S. mediterranea	Salami_Section2	SRR12426760	8476769
S. mediterranea	Salami_Section1	SRR12426761	5886612
L. stagnalis	Control1	SRR12675210	27713142
L. stagnalis	Control2	SRR12675206	25417785
L. stagnalis	Control3	SRR12675247	31242967
L. stagnalis	Control4	SRR12675229	19960951
L. stagnalis	Wounded1	SRR12675230	27023963
L. stagnalis	Wounded2	SRR12675245	23379402
L. stagnalis	Wounded3	SRR12675236	19302665
L. stagnalis	Wounded4	SRR12675227	14728302

Supplemental Table 2. Overview of Data Libraries. Summary of libraries used in data analysis of both Mollusca and outgroup species.

bioRxiv preprint doi: https://doi.org/10.1101/2025.05.19.654968; this version posted May 23, 2025. The copyright holder for this preprint (which was not certified by peer review) is the author/funder, who has granted bioRxiv a license to display the preprint in perpetuity. It is made available under aCC-BY 4.0 International license.

	Cgigas	Cvirginica	Hrufescens	Myessoensis	Obimaculoides	Rvenosa	Lstagnalis
**Bantam**	1	0	0	1	1	2	8
**Let-7**	1	2	1	0	1	1	10
**Mir-1**	1	1	1	2	0	1	12
**Mir-2**	1	5	2	12	5	16	84
**Mir-7**	1	1	0	2	1	1	6
**Mir-8**	1	1	1	1	1	2	10
**Mir-9**	1	1	0	1	1	1	9
**Mir-10**	4	1	1	2	1	2	31
**Mir-12**	1	1	1	1	0	2	9
**Mir-22**	1	1	1	1	1	3	19
**Mir-29**	1	4	1	2	2	4	19
**Mir-31**	1	0	1	1	2	3	9
**Mir-33**	1	0	0	0	2	1	8
**Mir-34**	1	1	1	1	0	3	10
**Mir-67**	1	3	0	3	1	1	12
**Mir-71**	1	2	1	2	1	2	10
**Mir-76**	1	2	1	1	1	2	9
**Mir-87**	1	2	2	1	1	2	12
**Mir-92**	1	4	6	5	1	7	34
**Mir-96**	1	2	1	1	1	2	25
**Mir-124**	1	1	1	1	1	3	12
**Mir-133**	1	1	0	1	1	1	11
**Mir-137**	1	2	0	2	1	2	10
**Mir-153**	1	1	1	1	1	3	10
**Mir-184**	1	1	2	7	2	1	18
**Mir-190**	1	1	0	1	0	1	9
**Mir-193**	1	2	1	1	1	2	8
**Mir-210**	1	1	0	1	3	2	5
**Mir-216**	1	1	1	1	2	2	17
**Mir-219**	0	1	1	1	2	3	7
**Mir-242**	1	0	1	1	0	1	0
**Mir-252**	1	1	0	1	1	4	13
**Mir-277**	1	2	1	2	1	3	9
**Mir-278**	1	2	1	1	1	6	10
**Mir-279**	1	2	1	1	1	2	10
**Mir-281**	0	1	1	2	1	0	14
**Mir-315**	1	1	0	1	2	2	10
**Mir-317**	1	2	0	1	0	2	11
**Mir-375**	1	4	0	5	1	0	16
**Mir-750**	1	0	0	1	1	1	9
**Mir-1175**	1	1	0	1	1	1	8
**Mir-1984**	1	1	0	3	3	1	8
**Mir-1986**	1	1	1	1	2	0	11
**Mir-1989**	1	0	0	1	0	1	0
**Mir-1990**	1	1	1	1	0	2	9
**Mir-1992**	1	0	2	1	1	2	10
**Mir-1993**	1	2	1	1	1	2	7
**Mir-1994**	1	2	2	1	1	4	20
**Mir-2001**	1	1	0	2	0	4	6
**Mir-2722**	0	0	1	1	1	2	7

Supplemental Table 3: Overview of miRNA Families in Mollusca. The top 50 conserved miRNA sequences identified amongst the mollusks examined.
